# Developing and validating with foot-care abilities questionnaire (F-CAQ) in a low risk population

**DOI:** 10.1186/1757-1146-8-S2-P7

**Published:** 2015-09-22

**Authors:** Tina Loxley

**Affiliations:** 1 Barwon Health Podiatry Department, University Hospital Geelong, Geelong, Victoria, 3216, Australia

**Keywords:** Foot-Care, Self-management, ASM, Podiatry

## Background

A validated instrument for measuring client's perceived problems with their abilities for foot-care was required to measure outcomes of the Meet Your Feet (MYF) program. The MYF pilot program is a component of the demand management strategy and linked with the Active Service Model (ASM) at Barwon Health Community Health and Rehabilitation Services. A literature search revealed no such instrument was available. We developed a questionnaire and tested it with a small sample group.

## Methods

A 17 item self-administered questionnaire, the foot-care abilities questionnaire (F-CAQ) was developed.

Face and content validity was established using the content validity index.

Ethics approval was obtained to involve low risk participants in testing the problems with F-CAQ. Data was analysed using the statistical package for Social Sciences (SPSS). Cronbach's alpha was used to measure internal consistency. Pearson correlation coefficients were used to examine the test-retest reliability of the F-CAQ items.

## Results

Nine health professionals, six clients and three lay people provided feedback about face and content validity. Items that rated 7/9 or higher, were kept. Seventeen of nineteen items were retained, seven were reworded and two rechecked for relevance.

The F-CAQ had Flesch readability ease of 82.4 and Flesch Kincaid grade level of 5.4.

Twenty-nine low risk clients participated in testing the reliability of the F-CAQ: age range 65-90, median age bracket 70s, mean age 75; 15 females and 14 males.

Complete data was available for 20 participants, used in reliability analyses.

The Cronbach's alpha score of .903 indicated a high degree of internal consistency. The Pearson correlation score of .855 indicated good stability of measurement over time.

## Conclusions

The F-CAQ has been proven to have face, content, internal consistency and test re-test reliability from a small sample group. Once this validated and reliable questionnaire development process is complete, use of the instrument pre and post the MYF Program will allow publication of outcomes from the MYF program. Future positive testing outcomes will allow the use of the validated and reliable F-CAQ to test outcomes from other population groups or in one to one podiatry interventions where the ASM is paramount.

